# Effect of a Hybrid Pumice–Portland Cement Extract on Corrosion Activity of Stainless Steel SS304 and Carbon Mild Steel A36

**DOI:** 10.3390/ma17102255

**Published:** 2024-05-10

**Authors:** David Bonfil, Lucien Veleva, Jose Ivan Escalante-Garcia

**Affiliations:** 1 Center for Research and Advances Study (CINVESTAV), Applied Physics Department, Campus Merida, Merida 97310, Yucatan, Mexico; david.bonfil@cinvestav.mx; 2 Center for Research and Advanced Study (CINVESTAV), Campus Saltillo, Ramos Arizpe 25900, Coahuila, Mexico; ivan.escalante@cinvestav.edu.mx

**Keywords:** carbon steel, stainless steel, hybrid cement, cement extract solution, pumice, corrosion potential, pH, SEM–EDS, XPS, EIS

## Abstract

The change in the corrosion activities of SS304 and the carbon steel A36 were studied during their exposure for 30 days to hybrid pumice-Portland cement extract (CE), to simulate the concrete–pore environment. The ionic composition and the initial pH (12.99) of the CE were influenced by the reduction of Portland cement (PC) content, volcanic pumice oxides and alkaline activators. Because of the air ${\mathrm{CO}}_{2\;}$ dissolution, the pH decreased and maintained a constant value ≈ 9.10 (established dynamic ionic equilibrium). The CE promoted the passivation of both steels and their free corrosion potential (OCP) reached positive values. On the surfaces, Fe and Cr oxides were formed, according to the nature of the steel. Over the time of exposure, the presence of chloride ions in the pumice caused a localized pitting attack, and for carbon steel, this fact may indicate an intermediate risk of corrosion. The chloride effect was retarded by the accumulation of ${{\mathrm{SO}}_{4}}^{2-}$ ions at the steel surfaces. Based on electrochemical impedance (EIS), the polarization resistance (Rp) and the thickness of the passive layers were calculated. Their values were compared with those previously reported for the steels exposed to CEs of Portland and supersulfated cements, and the hybrid cement was considered as a PC “green” alternative.

## 1. Introduction

The demand of PC has surged in recent years due to global urbanization to approximately 4 billion ton/year, representing 8–10% of the global ${\mathrm{CO}}_{2}$ emissions [1,2,3]. The ${\mathrm{CO}}_{2}$ emissions result almost in equal parts from the chemical decomposition of limestone into lime (${\mathrm{CaCO}}_{3}\to \mathrm{CaO}$) [4] and the use of fossil fuels. To address this environmental impact, various alternative “green” cements were developed, like blended, alkali-activated, supersulfated, and hybrid cements, among others. These cements involve the partial or complete replacement of the PC [5,6] with supplementary cementitious materials (SCM), such as natural pozzolan (volcanic materials), limestone, blast furnace slag, fly ash, calcinated clays and waste glass [7,8]. These alternative cements offer advantages including lower ${\mathrm{CO}}_{2}$ emission compared to PC, good mechanical strength, thermal resistance, among others [9,10,11,12].

Volcanic pumice, a natural pozzolan rich in ${\mathrm{SiO}}_{2}$ and ${\mathrm{Al}}_{2}O_{3}$ that is liable to dissolve in alkaline environments, was used as a precursor in supersulfated [13] and hybrid cements [14], along with various combinations of activators. Pumice-based hybrid cements [14] have shown promising compressive strength of ≈38.8–66.6 MPa after 28 and 90 days, due to the formation of C-S-H, C-A-S-H and ettringite. The composition of a hybrid cement labeled as “HB1” in a recent report [14] reduces the PC consumption by 50% and its manufacture involves low ${\mathrm{CO}}_{2\;}$ emissions of 0.15–0.30 kg ${\mathrm{CO}}_{2\;}$ per kg of binder. However, the application of alternative cements in concrete structures requires a careful characterization of the electrochemical corrosion behavior of the steel reinforcement, due to the modifications in the alkalinity and ion composition of the pore solution relative to the PC [15]. The alkaline environment of conventional PC concrete, with a pH ranging from 13 to 13.8, derives from the hydration of PC alkali oxides ($K_{2}O$, NaO), and ${\mathrm{Ca}(\mathrm{OH})}_{2}$ from the silicates hydration [4]. This alkaline environment encourages the formation of a protective (thin) corrosion layer of Fe (oxides/hydroxides) on the carbon steel surface, known as passive film, which prevents the corrosion progress, as long as an alkaline pH prevails [16]. Stainless steel, on the other hand, forms a passive film composed of an anhydrous inner layer rich in Cr oxides and outer Fe (oxides/hydroxides) [17,18]. The passive state of the reinforcement steels may be affected by the pH shifts towards less alkaline values [19]. Therefore, the reduction of the clinker factor and the use of SCM in alternative cements could significantly lower the pH of the pore solution [20,21], affecting the passive state of the reinforcement steels. For instance, the replacement of PC with 10% of silica fume may decrease the alkali content by ≈60% [22], due to the pozzolanic activity. On the other hand, depending on the alternative cement composition, the concrete pore solution may increase the concentration of the Na, Al, Si, and S ionic species [23,24,25,26], which would affect the ionic strength and resistivity of the bulk solution.

Investigations into the passivation behavior of steel in concrete pore solution environments have typically used model solutions to simulate the alkaline conditions. Model solutions prevent experimental difficulties for the study of the corrosion of steels embedded in concrete such as cell designs, position of auxiliary and reference electrodes, drop potential (IR) and its compensation, constraint of $O_{2}$ diffusion, among others. Saturated ${\mathrm{Ca}(\mathrm{OH})}_{2}$ (pH ≈ 12.6), NaOH and KOH solutions, or mixtures of them are commonly used as model pore solutions [27,28,29,30,31,32,33]. On the other hand, CE solutions were proposed as more representative alternatives to model solutions, offering a closer approximation of the chemical composition of concrete pore environments [34,35,36]. CE from a Type I cement paste with a water/cement ratio of =0.42, containing NaOH, KOH, ${\mathrm{Ca}(\mathrm{OH})}_{2}$ and ${\mathrm{CaSO}}_{4}\cdot H_{2}O$, formed a more protective passive layer than a saturated ${\mathrm{Ca}(\mathrm{OH})}_{2}$ solution [27], although the composition of any of the passive films was independent of pH.

During the immersion of an FeE500 mild steel in a model saturated with ${\mathrm{Ca}(\mathrm{OH})}_{2}$ (pH = 12.5), 0.1 NaOH (pH = 12.8) and 25 mM ${\mathrm{NaHCO}}_{3}/{\mathrm{Na}}_{2}{\mathrm{CO}}_{3}$ (pH = 10) solutions, the nature of the Fe oxide layers (${\mathrm{Fe}}^{3+}$ and ${\mathrm{Fe}}^{2+}$) was independent of the electrolyte in 10<pH<13; however, in the presence of carbonates, silica and sulfates (to simulate a carbonated pore environment), the decreased corrosion current was ascribed to the incorporation of the silica in the steel passive layer [37]. On the other hand, for Q235 mild steel (China manufacture) immersed in saturated ${\mathrm{Ca}(\mathrm{OH})}_{2}$ and NaOH (0.032 M, 0.1 M and 1 M, 12.5<pH<14), the nature of the passive films were similar (${\mathrm{Fe}}_{3}O_{4}$, ${\mathrm{Fe}}_{2}O_{3}$ and FeOOH); however, the thickness and the charge transfer resistance ($R_{\mathrm{ct}}$) were greater with increasing OH^−^ concentration [38]. An investigation on the electrochemical response of Fe electrodes immersed in saturated Ca(OH)_2_ and NaOH suggested that the inner passive layer was similar to Fe_3_O_4_, while the outer was a gelatinous Fe oxide, where the reversible ${\mathrm{Fe}}^{3+}/{\mathrm{Fe}}^{2+}$ reaction occurs [39]. The corrosion inhibition in saturated ${\mathrm{Ca}(\mathrm{OH})}_{2}$ is explained by ${\mathrm{Ca}}^{2+}$ ion adsorption on the outer passive film, producing a gelatinous water, leading to the formation of protective oxides [40]. Another study indicated that the addition of sulfates (${{\mathrm{SO}}_{4}}^{2-}$) [41] increases the ionic strength of the pore solution environment but decreases its buffering capacity, leading to the corrosion of a mild steel FeE500 under a high carbonate alkalinity, dissolving the passive film formed on low-carbon steel HRB335 [42] and ER316L black steel [19], immersed in saturated ${\mathrm{Ca}(\mathrm{OH})}_{2}$ [42]. A study on the influence of a sulfate salt type on the passive film stability of carbon steel, exposed to saturated ${\mathrm{Ca}(\mathrm{OH})}_{2}$, revealed that ${\mathrm{MgSO}}_{4}$ and ${\left({\mathrm{NH}}_{4}\right)}_{2}{\mathrm{SO}}_{4}$ led to higher corrosion rates than ${\mathrm{Na}}_{2}{\mathrm{SO}}_{4}$ [43]. Other results demonstrated that the pH of the pore solution increased slightly in the presence of ${\mathrm{Na}}_{2}{\mathrm{SO}}_{4}$, while ${\mathrm{MgSO}}_{4}$ and ${\left({\mathrm{NH}}_{4}\right)}_{2}{\mathrm{SO}}_{4}$ reduced the pH, thus reducing the effectiveness of the passive layer, which is enhanced in the presence of chloride ions [44,45]. Also, the immersion of a carbon steel Q235 in a simulated alkali-activated slag with sodium silicate slag (pH ≈ 13.35–13.64) showed the formation of a compact microstructure aluminate/silicate layer on the steel surface because of the reduced oxygen content derived from oxidation of the $S^{2-}$ species [46]. However, at a high S content (≈10,863 mg/L), FeS was detected, reducing the Fe^2+^:Fe^3+^ ratio, which led to a more porous passive film with lower corrosion resistance, evidenced by the very negative OCP (≈−0.55 V/SCE).

This study explores the effect of a new “green” hybrid cement, labeled as “HB1” [14], with reduced PC content, replaced by volcanic pumice (45.82%), on the corrosion activity of mild carbon steel A36 and stainless steel 304, as reinforcement in concrete, when exposed to the CE of “HB1”, as a model solution closer to the chemical composition of a concrete–pore environment. The pH of the CE solution and the steel OCP, considered as free corrosion potential, were registered over 30 days. To characterize the interface of the steel–CE, EIS measurements were also performed. The steel surfaces were characterized using scanning electron microscopy–energy dispersive spectroscopy (SEM–EDS) and X-ray photoelectron spectroscopy (XPS). The results were compared to those obtained from exposure of CEs to Portland [47] and of supersulfated [48] cements, considered as a “green” alternative for the partial replacement of PC. To the best of our knowledge, this is the first investigation with this approach.

## 2. Materials and Methods

### 2.1. Steel Samples

Flat samples of austenitic stainless steel 304 (Outokumpu Mexinox, San Luis Potosí Mexico) and mild carbon steel A36 (Steeland, Guadalajara, Mexico) were cut to dimensions of 2 × 2 cm and thickness 0.1 cm. The surfaces were abraded with 4000 grit SiC paper using ethanol as lubricant, sonicated for 10 min (Branson 1510, Branson Ultrasonics Co., Danbury, CT, USA) and dried at 21 °C. The steel compositions (wt.%) are presented in Table 1.

### 2.2. Hybrid Cement “HB1” and Its Cement Extract Solution

Table 2 presents the composition of “HB1” hybrid cement [14], i.e., pumice, PC (CPC30R) as precursors, industrial grate powders of ${\mathrm{Na}}_{2}{\mathrm{SO}}_{4}$ and calcium hydroxide ${\mathrm{Ca}(\mathrm{OH})}_{2}$ as alkaline activators, in a molar ratio ${\mathrm{Na}}_{2}{\mathrm{SO}}_{4}/{\mathrm{Ca}(\mathrm{OH})}_{2}=1$.

Table 3 presents the composition of the “SS1” supersulfated cement, previously reported [48].

The hybrid cement “HB1” contains a higher proportion of PC (CPC30R) and lower pumice contents than the “SS1” (Table 2 and Table 3). However, the “HB1” involves ${\mathrm{Na}}_{2}{\mathrm{SO}}_{4}$ and ${\mathrm{Ca}(\mathrm{OH})}_{2}$ as alkaline activators, while the “SS1” uses CaO as the alkaline activator and hemihydrate as the sulfatic activator of [13]. These composition differences promote distinct reactions during the hydration process and thus, originating variations in the chemical composition of the CE concrete pore solutions. It is reported that the alkaline activators, as a part of the “HB1”, react in situ (Equation (1)) forming ${\mathrm{CaSO}}_{4}$ and NaOH [14], both activators of the pumice [13]. Notably, NaOH would contribute to the alkalinity of the concrete pore solution.

(1) $${\mathrm{Na}}_{2}{\mathrm{SO}}_{4}+{\mathrm{Ca}(\mathrm{OH})}_{2}\to {\;\mathrm{CaSO}}_{4}+\mathrm{NaOH}$$

Table 4 compares the chemical oxide composition of hybrid cement “HB1” with those of two previously reported cements, i.e., supersulfated cement “SS1” [13,48] and PC [49].

The main oxides of the hybrid cement “HB1” are CaO (29.78%) and ${\mathrm{SiO}}_{2}\;$(43.05%). The CaO content in PC (58.42%) is about two-fold that of “HB1” [49], while the inverse can be noted regarding ${\mathrm{SiO}}_{2}$ contents, due to the pumice contribution. Additionally, “HB1” presents greater contents of ${\mathrm{Al}}_{2}O_{3}$, $K_{2}O$, ${\mathrm{SO}}_{3}$ and ${\mathrm{Na}}_{2}O$ than PC; the last two are attributed to the ${\mathrm{Na}}_{2}{\mathrm{SO}}_{4}$ used as activator. On the other hand, the ${\mathrm{SO}}_{3}$ and ${\mathrm{Na}}_{2}O$ contents are different among “HB1” and the supersulfated cement “SS1”, attributable to differences in the type and amounts of activators used.

The cement extract was prepared with the “HB1”cement and ultrapure deionized water (18.2 MΩ cm) in a water/cement ratio = 1. The mixture was stirred and left during 24 h for the cement to react (hydrate) in a closed recipient. The supernatant was then filtered (2.5 µm pore size filter paper, Whatman, Kent, UK); to avoid carbonation the solution was kept in a sealed container. Table 5 presents the chemical composition of the “HB1” CE solution obtained by absorption spectrometry and atomic emission by plasma and ion selective electrode for the free ${\mathrm{Cl}}^{-}$ ions.

The main ions present are ${\mathrm{Na}}^{+}$, ${{\mathrm{SO}}_{4}}^{2-}$, $K^{+}$, ${\mathrm{Ca}}^{2+}$ and ${\mathrm{OH}}^{-}$. The alkalinity of the hybrid cement (pH = 12.99) is contributed to the high contents of ${\mathrm{Na}}^{+}$ and $K^{+}$ (NaOH and KOH alkaline hydroxides formation), and that of ${\mathrm{Ca}}^{2+}$ (Ca(OH)_2_ formation). According to [26], the solubility of the ${\mathrm{Ca}}^{2+}$ increases in the presence of ${{\mathrm{SO}}_{4}}^{2-}$ ions and this fact is attributed to the high pH = 12.99 and ionic strength of the pore solution. On the other hand the presence of chloride ions are originated from the pumice [14].

### 2.3. Immersion Test

The immersion tests were carried out according to the ASTM-NACE/ ASTM G31-12a standard [50]. The steels with 0.8 ${\mathrm{cm}}^{2}$ of working area were immersed in 10 mL of “HB1” CE, over 30 days, in sealed containers (using paraffin tape). At the periods of 7 and 30 days, the samples were withdrawn, and air dried at 21 °C. The pH of the CE solution was measured (PH60 Premium Line, pH tester, Apera Instruments, LLC., Columbus, OH, USA) after each period of exposure. The steel surfaces of the withdrawn samples were analyzed using SEM–EDS (XL–30 ESEM-JEOL JSM-7600F, JEOL Ltd., Tokyo, Japan) and XPS (K-Alpha, Thermo Scientific, Waltham, MA, USA) at different times of erosion with a scanning Ar-ion gun, was used to identify the corrosion products. To observe the attack on the surface, the formed layers were removed [51], and the steel surfaces were characterized by SEM–EDS.

### 2.4. Electrochemical Measurements

Electrochemical measurements were performed with a typical three-electrode configuration (inside a Faraday cage): the steel flat samples of A36 and 304 (of 4 m^2^ area), a saturated calomel electrode (SCE) and a Pt mesh, were the working, reference and auxiliary electrodes, respectively. The potentiostat used for the electrochemical experiments (294 K) was the Interface-1000E potentiostat/galvanostat/ZRA, Gamry Instruments, Philadelphia, PA, USA. The change of the OCP of the steel electrodes over time was monitored and considered as free corrosion potential. EIS at OCP was measured from 100 kHz to 10 mHz frecuency range, with an alternating current (AC) signal of ±10 mV and a sampling size of 10 data points per decade. EIS diagrams (Nyquist and Bode) were obtained at 1, 7, 14, 21, and 30 days of immersion. The data were analyzed by Gamry Echem Analyst software^®^ (version 7.1, Philadelphia, PA, USA) and all tests were performed in triplicate.

## 3. Results and Discussion

### 3.1. Change in Time of pH of the “HB1” CE Solution and OCP of the Steels

Table 6 presents the change in time of the “HB1” CE solution’s pH and OCP (vs. SHE, Saturated Hydrogen Electrode), during the exposure to stainless steel 304 and A36 carbon steel of up to 30 days.

The initial pH of the CE extract solution was 12.99, which is similar to that of Portland CE (pH≈13) due to the contribution of the greater contents of $K_{2}O$ and ${\mathrm{Na}}_{2}O$ (volcanic pumice) and the reaction of the activators (Equation (1)) to the alkalinity [13,14]. The pH of the CE solutions tended towards less alkaline values of ≈9.59 at 14 days, and then the pH kept an almost constant value ≈ 9.10 up to the 30 days, indicating the establishement of dynamic ionic equilibrium. Studies suggest that the formation of the weak carbonic acid (${\mathrm{CO}}_{2}$ air dissolution in aqueous solutions) may act as a buffer for the pH change [52,53,54]. The carbonate ions may react with the ${\mathrm{Ca}}^{2+}$, $K^{+}$, ${\mathrm{Na}}^{+}$ cations (Table 5) forming their carbonates, of which only the calcium carbonate precipitates (${\mathrm{CaCO}}_{3}$) in its more common crystalline polymorphs, such as calcite and vaterite, depending on the concrete pore solution [55]. Even though the pH dropped, the OCP value of stainless steel 304 (−204.899 mV) turned to more positive/noble ones and reached +265.085 mV at 30 days of exposure, indicating that the steel was passivated during the immersion time. The OCP of carbon steel A36 (−89.053 mV) also tended to more positive values, reaching +88.18 mV, in an attempt to obtain a passive state, although this OCP value may indicate an intermediate risk of corrosion, according to the ASTM C876 [56].

### 3.2. Stainless Steel 304 Surface Characterization after Immersion in the “HB1” CE Solution 

**Figure 1:**
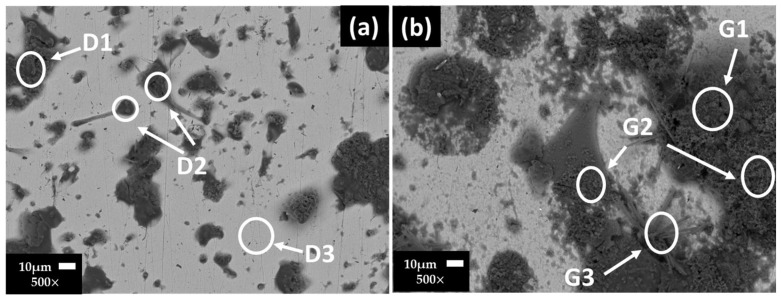
Stainless steel 304 SEM images after (**a**) 7 and (**b**) 30 days of exposure to “HB1” CE solution.

Figure 1 shows the SEM images of the stainless steel 304 surfaces and their EDS analysis (Table 7) after 7 (Figure 1a) and 30 (Figure 1b) days of exposure to the “HB1” CE solution. After 7 days (at OCP = +128.126 and pH = 10.36), the EDS analysis presented for zones D1 and D2 show a high content of O, S, Na, K and Ca (ascribed to the precipitates of sulfates and carbonates). Zone D3 corresponds to the matrix of the stainless steel (Figure 1a). According to the more positive value of OCP (+265 mV) at 30 days, the stainless steel improved its passive state (Table 6) and a more extended surface layer was observed (Figure 1b), formed mainly by sulfate and carbonate precipitates (zones G1, G2, G3, Table 7). The low content of Cr was ascribed to Cr oxide. On the other hand, traces of Cl were detected, suggesting that ${\mathrm{Cl}}^{-}$ anions may accumulate over time at the metal–solution interface inside the pits (localized corrosion attacks); however, they will not be able to grow any further, and the sulfate ions would act as pitting inhibitors [57].

**Figure 2:**
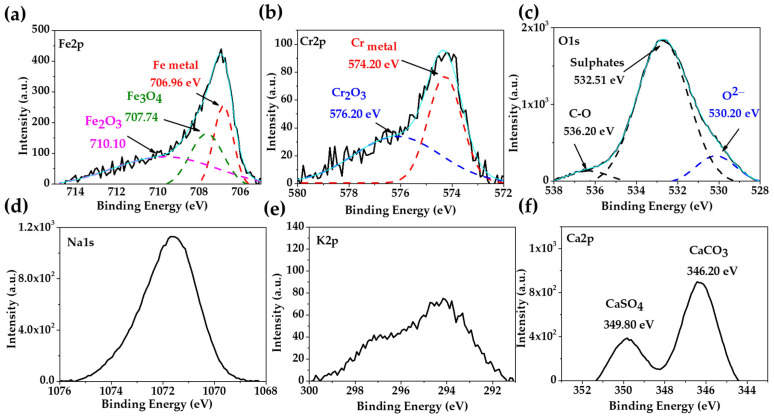
XPS spectra of stainless steel 304 exposed for 30 days to “HB1” CE solution: (**a**) Fe2p; (**b**) Cr2p; (**c**) O1s; (**d**) Na1s; (**e**) K2p; (**f**) Ca2p; the light blue line corresponds to the envelope.

Figure 2 presents the XPS spectra of the stainless steel 304 after 30 days of exposure to the “HB1” CE solution. The deconvolution of the Fe2p (Figure 2a), Cr2p (Figure 2b) and O1s (Figure 2c) peaks may suggest that the passive layer was composed of ${\mathrm{Fe}}_{3}O_{4}$ (707.74 eV), ${\mathrm{Fe}}_{2}O_{3}$ (710.10 eV) and ${\mathrm{Cr}}_{2}O_{3}$ (576.20 eV) [58,59,60]. The different chemical states of the O1s were idendified and ascribed to oxides (530.20 eV), sulphates (532.51 eV), and in a low intensity the peak of C-O (536.20 eV), associated with the peaks of Na (Figure 2d), K1s (Figure 2e) and Ca1s (Figure 2f) may be atributed to carbonates or sulphates [61,62]. The reported results have demonstrated that, at an alkalinity of pH 9, the passive layer of stainless steel is enriched in chromium species, of which, the most stable is ${\mathrm{Cr}}_{2}O_{3}$ [58,63], which likewise enriches the more oxidized iron species of Fe^3+^ (${\mathrm{Fe}}_{2}O_{3}$) due to the decomposition of magnetite (${\mathrm{Fe}}_{3}O_{4}$) [17]. These facts are in accordance with the dual-layer structure of the passive film of stainless steel, composed of an inner stable Cr oxide layer, and an external layer of Fe oxides/hydroxides [35,36,64,65].

### 3.3. Carbon Steel A36 Surface Characterization after the Immersion in the “HB1” CE Solution 

**Figure 3:**
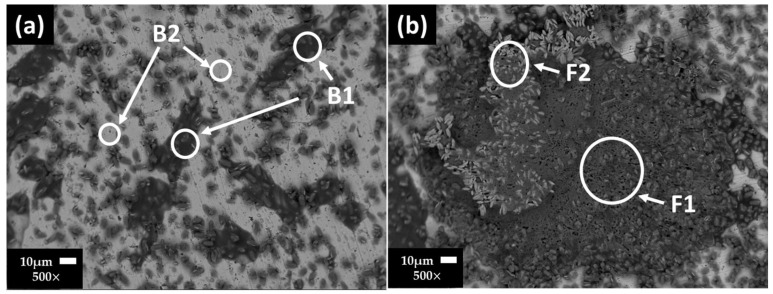
Carbon steel A36 SEM images after (**a**) 7 and (**b**) 30 days of exposure to “HB1” CE solution.

Figure 3 shows the SEM images of carbon steel A36 surfaces and their EDS analysis (Table 8) after 7 (Figure 3a) and 30 (Figure 3b) days of exposure to the “HB1” CE solution. At 7 days, a non-homogeneous layer of precipitates was observed: high content of O, Na, S, followed by Ca and at lower contents were C and K (zone B1), considered to be part of the sulfates and carbonates; zone B2 was ascribed to the matrix of the carbon steel. The presence of corrosion products was not contemplated due to the positive steel OCP value (≈+48.58 mV, at pH = 10.11). After 30 days of exposure (at OCP ≈ +88.18 mV and pH = 9.10), on the A36 surface, more extended layers, composed mainly of carbonates and sulfates, were observed along with a lower content of Fe oxides, according to the EDS analysis (Table 8).

**Figure 4:**
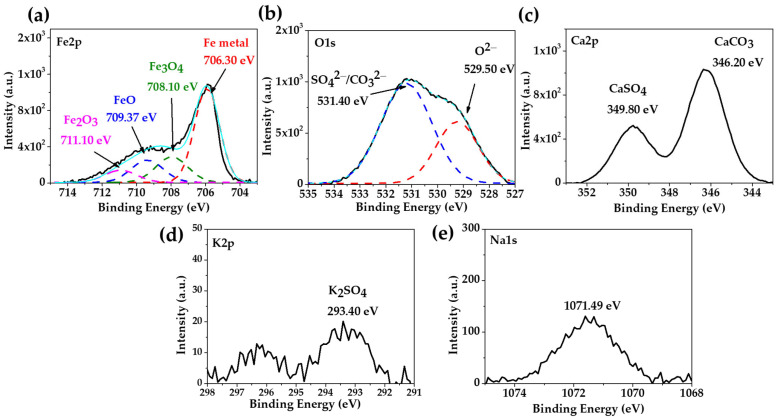
XPS spectra of carbon steel A36 exposed to “HB1” CE solution for 30 days: (**a**) Fe2p; (**b**) O1s; (**c**) Ca2p; (**d**) K2p; (**e**) Na1s; the light blue line corresponds to the envelope.

The XPS spectra of the carbon steel A36 after 30 days of exposure to the “HB1” CE solution are presented in Figure 4. The deconvolution of the Fe2p peak (Figure 4a) and that of O1s (Figure 4b) were associated with the Fe metal (706.30 eV) as a part of the metal matrix, while ${\mathrm{Fe}}_{3}O_{4}$ (708.10 eV), FeO (709.37 eV) and ${\mathrm{Fe}}_{2}O_{3}$ (711.10 eV) corresponded to the passive film [66]. The two peaks of deconvoluted O1s (Figure 4b) were ascribed to the oxides $O^{2-}$ (529.5 eV), carbonates and sulphates ${{\mathrm{SO}}_{4}}^{2-}/{{\mathrm{CO}}_{3}}^{2-}$ (531.40 eV) [61,62]. An investigation reported that in a highly alkaline environment with a pH ≈ 13, the formation of magnetite ${\mathrm{Fe}}_{3}O_{4}$ is promoted, indicating the transition to the passive state [67]. The presence of magnetite in the passive layer performs an essential role in steel corrosion protection (passivation) and is in accordance with the positive shift of the OCP (Table 6). However, when the pH decreases ≈ 9, the magnetite decomposes forming ${\mathrm{Fe}}^{2+}$ and ${\mathrm{Fe}}^{3+}$ oxides or hydroxides, depending on the grade of oxidation [17,68], accompanied by an increase in the film thickness [69]. The peaks of Ca2p (Figure 4c), K2p (Figure 4d) and Na1s (Figure 4e) were attributed to the different crystals (Figure 3b, Table 8).

### 3.4. Steel Surface Deterioration after the Immersion Tests in “HB1” CE Solution

**Figure 5:**
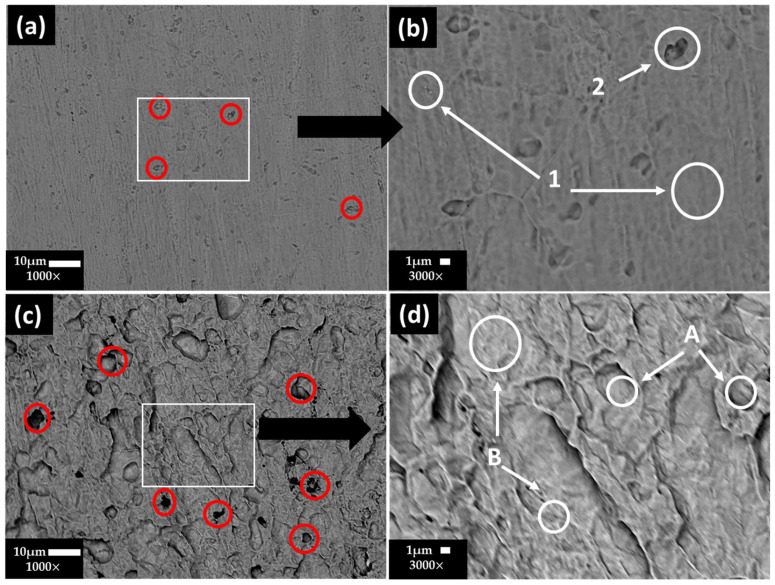
SEM images of stainless steel 304 surface: (**a**) ×1000 and (**b**) ×3000 magnification, and carbon steel A36: (**c**) ×1000 and (**d**) ×3000 magnification, after removal of the layer formed during the exposure for 30 days to “HB1” CE solution.

Figure 5 shows the SEM images of the stainless steel 304 surface at (a) ×1000 and (b) ×3000 and the carbon steel A36 surface at (c) ×1000 and (d) ×3000 after the chemical removal of the layers formed along the exposure to the “HB1” CE solution for 30 days. On the stainless steel 304 surface (Figure 5a,b), some small pits (labeled as red circles) were observed, associated with the ${\mathrm{Cl}}^{-}$ ions present in the “HB1” CE solution (172 mg/L, Table 5), which penetrated through the pores of the passive protective film of ${\mathrm{Cr}}_{2}O_{3}$ and ${\mathrm{Fe}}_{3}O_{4}$, as a consequence of their small size [70]. In accordance with the EDS analysis (Table 9), Fe, Cr and Ni (zones 1–2) are the main constituents, attributed to the Fe–Cr–Ni crystal structure of the steel. The presence of C and Mn in zone 2 could be ascribed to MnC precipitates and Fe carbides, acting as local cathodes [71], in which vicinity the small pits are observed (Figure 5a, red circles). The content of Ni contributes to the resistance of steel to pitting corrosion.

On the other hand, for the carbon steel A36 (Figure 5c,d), the EDS analysis showed that the steel matrix was composed mainly of Fe and a low content of C (zones A and B), and the local Fe carbides may act as active cathodes, in which vicinity the pits (localized corrosion) occurred (Figure 5c, red circles) because of the presence of ${\mathrm{Cl}}^{-}$ ions in the “HB1” CE solution (172 mg $L^{-1}$ Table 5), which penetrated through the passive film defects or partial dissolution of Fe oxides due to the drop in the pH of the CE solution. However, in the presence of ${{\mathrm{SO}}_{4}}^{2-}$ (10,115 mg $L^{-1}$, Table 5), with a higher charge than that of ${\mathrm{Cl}}^{-}$ ions, ${{\mathrm{SO}}_{4}}^{2-}$ will be preferentially accumulated at the metal–CE pore solution interface, causing the reduction of the potential inside the pit [57]. This fact could be considered as a retarding effect of the chloride attack, in accordance with the observed positive shift in the OCP potential (Table 6), although with a drop in the pH.

### 3.5. EIS Diagrams (Nyquist and Bode)

**Figure 6:**
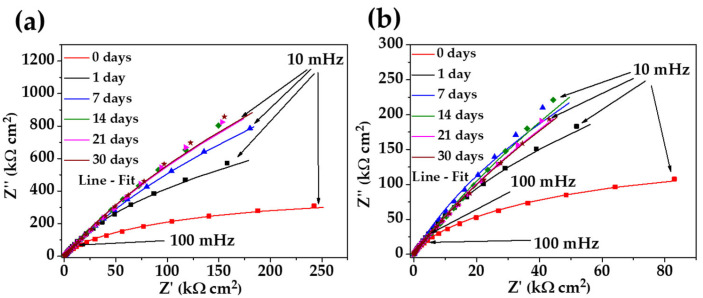
Nyquist plots of stainless steel 304 (**a**) and carbon steel A36 (**b**) with their fitting lines, after different times of exposure to the “HB1” CE solution for 30 days.

Figure 6 compares the Nyquist diagrams of stainless steel 304 (Figure 6a) with those of the carbon steel A36 (Figure 6b) during the exposure to the “HB1” CE solution for up to 30 days. Over the time of exposure, the diagrams of the stainless steel at the low-frequency range (10–100 mHz) displayed a semi-linear diffusion impedance with an increase in the imaginary value of impedance Z’’ up to ≈815 $k\Omega \;{\mathrm{cm}}^{2}$ at 30 days, suggesting the formation of a thickening passive layer on the surface, which is enriched with Cr species (${\mathrm{Cr}}_{2}O_{3}$) and Fe oxides (${\mathrm{Fe}}_{3}O_{4}$ and ${\mathrm{Fe}}_{2}O_{3}$), according to the XPS analysis. These facts agree with the displacement of the OCP to more positive/noble values (Table 6) because it is reported that the growth of ${\mathrm{Cr}}_{2}O_{3}$ is related to the formation of more dense and less conductive passive film (due to the filling of cation vacancies by Cr) [72]. Meanwhile, the Nyquist diagram of the carbon steel A36 (Figure 6b) also displayed a semi-linear diffusion impedance (at the low-frequency domain), with an increment in the impedance Z’’ value at up to 14 days (≈215 $k\Omega \;{\mathrm{cm}}^{2}$) because of the passive layer of ${\mathrm{Fe}}_{3}O_{4}$ (magnetite) on the steel surface, corroborating the tendency of the OPC to move to more positive values (Table 6). However, at the latter times (21 and 30 days), when the pH drops to ≈9.10, the Z’’ decreases slightly to ≈185 $k\Omega \;{\mathrm{cm}}^{2}$, associated with the partial decomposition of magnetite to iron species such as ${\mathrm{Fe}}_{2}O_{3}$ and FeO [17], accompanied by the effect of the chlorides ions that reached the surface causing a local depassivation of the steel.

**Figure 7:**
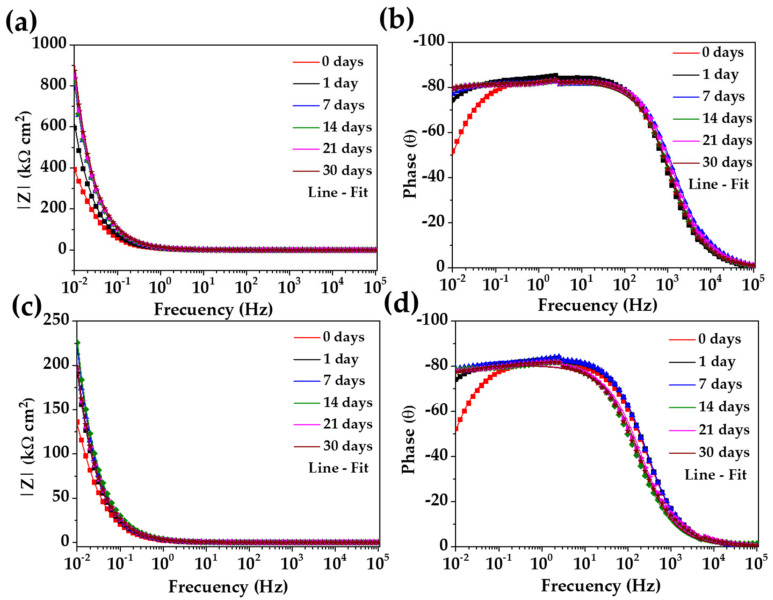
Bode plots of stainless steel 304 (**a**,**b**) and carbon steel B450C (**c**,**d**) with their fitting lines, after different times of exposure to the “HB1” CE solution for 30 days.

Figure 7 shows the Bode diagrams of the stainless steel 304 (a, b) and those of carbon steel A36 (c, d). The impedance module |Z| of stainless steel 304 (Figure 7a) confirmed its increase over time, meanwhile, the phase angle (θ) stabilizes to θ≈80° (Figure 7b), showing a passive film of ${\mathrm{Cr}}_{2}O_{3}$, ${\mathrm{Fe}}_{3}O_{4}$ and ${\mathrm{Fe}}_{2}O_{3}$ was formed that has capacitive behavior. That is, it accumulates electrical charges and increases the energy barrier required for the diffusion of aggressive species ($O_{2}$ and ${\mathrm{Cl}}^{-}$) from the CE solution to the steel interface occurring across the passive layer [72,73,74,75]. For the carbon steel A36, the tendency of the impedance module |Z| (Figure 7c) increment was influenced by the change in pH and the passive layer composition (as was mentioned above) and the phase angle reached θ≈77°, indicating the slightly lower capacitive nature of the passive film formed on the carbon steel surface. Even with these changes, A36 tended to passivate in the “HB1” CE solution, a fact confirmed by the OCP values (Table 6) and that of the phase angle (Figure 7d). Although both steels appear to be protected, it is evident that the impedance module values of the stainless steel 304 corrosion behavior were ≈3 times higher than those of the carbon steel A36 due to the specifics of the passive layers formed on both steel surfaces.

**Figure 8:**
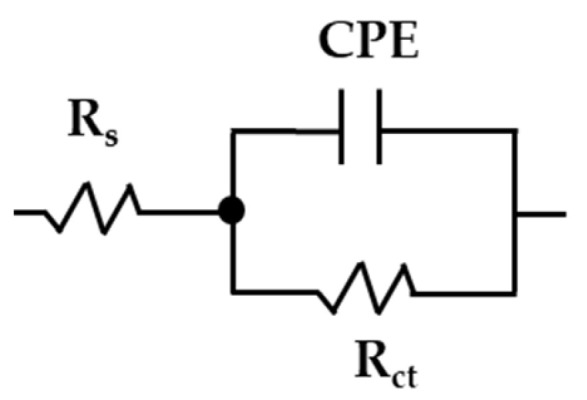
Equivalent circuit used for the stainless steel 304 and carbon steel A36 EIS data fit during the exposure to the “HB1” CE solution.

An equivalent circuit with only one time constant (simplified Randles, Figure 8) was used to quantify the EIS data and to describe the corrosion behavior of the studied steels during their exposure to the “HB1” CE solution [42,76,77]. The $R_{s}$ is the solution resistance at the steel–electrolyte interface (depending on the pH and ionic composition); $R_{\mathrm{ct}}$ is the charge transfer resistance; the constant phase element CPE was used instead of the double-layer capacitance in the presence of a passive layer, depending on its composition and porosity, as well as on the surface substrate roughness and distribution of anodic/cathodic active sites [78,79]. The interpretation of the constant phase element depends on the exponential factor value *n*, which ranges from 0 to 1: when the *n* tends to 0, the CPE behaves as a resistor, while the value of *n* tends to 1, it represents a capacitive behavior [42,80].

The fitting parameters obtained from the EIS measurements are presented in Table 10, and their goodness-of-fit χ^2^ ($10^{-4}$) was good in most cases. The values of *n* for stainless steel 304 and carbon steel A36 were relatively constant n ≈ 0.90 during the immersion of up to 30 days, confirming the capacitive behavior of the passive layers formed during the exposure to the “HB1” CE solution. The polarization resistance ($R_{p}$), as an almost equivalent of the charge transfer resistance $R_{\mathrm{ct}}$ values (minus solution resistance), was used as an indicator of the passive state’s stability of the studied steels, over the time of their exposure to the “HB1” CE solution. For stainless steel 304, the $R_{p}$ values increased almost 18 times after 30 days, reaching a stable value of ≈12,380 $k\Omega \;{\mathrm{cm}}^{2}$ because of the ${\mathrm{Cr}}_{2}O_{3}$ enrichment of the passive layer. For the carbon steel A36, the $R_{p}$ values also tended to increase by almost 16 times, reaching ≈4268 $k\Omega \;{\mathrm{cm}}^{2}$ at 14 days (at pH ≈ 9.10), decreasing slightly to $R_{p}$ ≈ 3186 $k\Omega \;{\mathrm{cm}}^{2}$ (at 30 days), being ≈4 times lower than that of the stainless steel 304.

The effective capacitance values C were calculated from the CPE values according to the Brug formula (Equation (2)) [80]. The C values were related to the passive layer thickness *d* formed on the studied steels by Equation (3) [81,82], where $\varepsilon_{0}$ is the vacuum permittivity ($8.85\times 10^{-14}\;F\;{\mathrm{cm}}^{-1}$), A is the working area (${\mathrm{cm}}^{2}$) and ε is the dielectric constant of the passive layer: 15.6 for stainless steel [64,83] and ≈12 for carbon steel [84].

(2) $$C={\mathrm{CPE}\;}^{\frac{1}{n}}{\left(\frac{R_{s}R_{\mathrm{ct}}}{R_{s}+{\;R}_{\mathrm{ct}}}\right)}^{\frac{1-n}{n}}$$

(3) $$d=\frac{\varepsilon \varepsilon_{0}A}{C}$$

**Figure 9:**
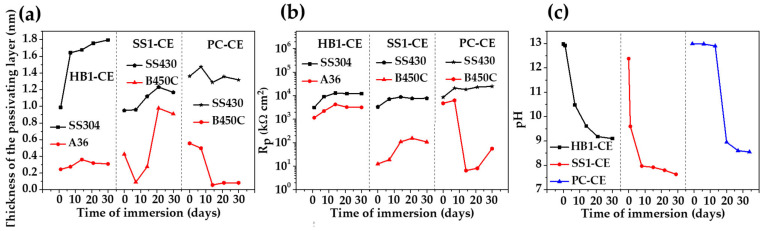
Evolution of the average passive layer thickness *d* (**a**) and $R_{p}$ values (**b**) of the stainless and carbon steels because of pH change during 30 days of immersion in (**c**) “HB1” (this study), “SS1-CE” [48] and “PC-CE” solutions [47]. Note: the EIS data of the compared CE solutions were fitted to the same proposed equivalent circuit, presented in Figure 8.

Figure 9 compares the evolution of the passive layer thickness (Figure 9a) and polarization resistance ($R_{p}$, Figure 9b) over the time of the exposure of the stainless steel 304 and the carbon steel A36 to the “HB1” CE solution. During this period, changes in pH occurred (Figure 9c). The values of these parameters were compared (Figure 9a–c) to those previously reported for stainless steel and carbon steel exposed to supersulfated (“SS1-CE”) [48] and Portland (“PC-CE”) cement extract solutions [47]. The thickness (*d*) of the passive layers formed on the stainless steel surface at 30 days of exposure was: d ≈ 1.8 nm in the “HB1” CE solution; d ≈ 1.3 nm in “PC-CE” and d ≈ 1.1 nm in “SS1-CE” (Figure 9a). This behavior may be associated with the pH of each CE solution over the period of 30 days (Figure 9b): the final pH ≈ 9.10 of the “HB1” CE promoted the formation of a thicker passive layer enriched with ${\mathrm{Cr}}_{2}O_{3}$ because of the ${\mathrm{Fe}}_{3}O_{4}$ partial decomposition (according to the XPS analysis) [84]. Meanwhile, the pH of the “PC-CE” dropped to ≈8.60 for 14 days (Figure 9c), and the passive layer presented corrosion products, such as ${\mathrm{Cr}(\mathrm{OH})}_{3}$, indicating the oxidation of ${\mathrm{Cr}}_{2}O_{3}$; on the other hand, the partial dissolution of Fe oxides at a lower pH was also suggested, and thus, the passive layer thickness *d* fluctuated due to passivation and depassivation processes (Figure 9a). During the exposure of the stainless steel to the “SS1-CE” solution, the pH dropped from the first day and reached a value of ≈7.8 (Figure 9c), when the passive layer was mainly composed of ${\mathrm{Cr}}_{2}O_{3}$, ${\mathrm{Cr}(\mathrm{OH})}_{3}$ and FeOOH corrosion products (probably associated with the magnetite decomposition), causing an increase in the layer thickness (Figure 9a), and from 21 days, started decreasing, probably because of the partial dissolution of the corrosion products (probably associated with the magnetite decomposition), causing an increase in the layer thickness (Figure 9a). The stainless steels presented higher values of polarization resistance ($R_{p}$, Figure 9b) in all CE solutions compared with those of the carbon steels, attributed mainly to the presence of capacitive properties of the ${\mathrm{Cr}}_{2}O_{3}$, with the minor values for the steel exposed to the “SS1-CE” solution, while the $R_{p}$ values were similar for the “PC-CE” and the “HB1” CE solutions.

The carbon steel A36 showed an average of thickness d ≈ 0.3 nm for the formed passive layer of (Figure 9a), during the exposure to the “HB1” CE solution (this study) and reached a value of polarization resistance Rp ≈ 3186 $k\Omega \;{\mathrm{cm}}^{2}$ at 30 days, being ≈1.5 order of magnitude higher than those values of $R_{p}$ of the passive films developed on the carbon steel B450C surfaces exposed to the “SS1-CE” extract and in the “PC-CE” solutions. These facts were ascribed to differences in the passive layer composition and its effect on the $R_{p}$: for example, one layer of corrosion products could be thicker but be less protective for the metal surface, and thus, have low $R_{p}$ values; this was the evidence presented by the carbon steel in the “SS1-CE” solution when the pH dropped to ≈7.6 at 30 days, causing active corrosion for the Fe matrix [48]. For the carbon steel B450C exposed to the “PC-CE” solution, the passive film tended to disappear after 14 days because of the drop in pH, reaching a value of ≈8.6 at 30 days, causing the initiation of active corrosion of the Fe matrix, forming a less protective layer (low values of $R_{p}$). In contrast, the high content of KOH and NaOH of the “HB1” CE solution (Table 5) contributed to the alkalinity of the concrete–pore solution, acting as an alkaline reserve, maintaining the pH ≈ 9.10. This environment facilitated the formation of protective passive films on stainless steel and carbon steel surfaces.

The comparative results (Figure 9) showed that the steel corrosion behavior is controlled by the chemistry of the CE solution, which simulates the concrete–pore solution and may influence its composition and alkalinity (pH), likewise modifying the thickness of the formed passive layer on the steel surface [85,86]. On the other hand, the nature of the steel is another very important factor that determines the initial passivation state and surface chemistry over the time of exposure to the concrete–pore environment. In a general view, the passive film formed on carbon steel in simulated concrete–pore solutions consisted of Fe oxides (${\mathrm{Fe}}^{2+}$ and ${\mathrm{Fe}}^{3+}$), including FeOOH, with a typical thickness of the passive layer in the range of 5 nm–13 nm [87], which was thicker in the solution with a higher pH value. The use of different alkaline activators may lead to distinct reactions during the cement hydration process and thus, originating variations in the chemical composition of the pore solution. In this aspect, the results of this study suggest that the “HB1” CE solution may promote the passivation of the carbon steel A36 and stainless steel 304, and thus, the hybrid cement “HB1” may be recommended as a substitute for Portland cement. In chloride environments (marine), the use of carbon steel or stainless steel as reinforcement in concrete based on the hybrid “HB1” cement will depend mainly on the needed service life of the structure, as well as on the nature of the steel used.

## 4. Conclusions

1. The change in the corrosion activities of SS304 and carbon steel A36 were studied during their exposure for 30 days to an aqueous extract solution of a hybrid cement, labeled as “HB1”, composed of CPC30R, partially replaced by volcanic pumice, in the presence of ${\mathrm{Na}}_{2}{\mathrm{SO}}_{4}$, ${\mathrm{Ca}(\mathrm{OH})}_{2}$ as alkaline activators.
2. The ionic composition of the “HB1” CE is influenced by the reduction of PC content, the composition of the volcanic pumice and the presence of alkaline activators.
3. The initial pH of the “HB1” CE solution was 12.99 (similar to that of the “PC–CE” solution) due to the contribution of the greater contents of $K_{2}O$ and ${\mathrm{Na}}_{2}O$ (volcanic pumice) and the reaction of the activators. The pH diminished and maintained an almost constant value ≈ 9.10 for the 30 days, indicating an established dynamic ionic equilibrium because of the air ${\mathrm{CO}}_{2}$ dissolution.
4. In the meantime, the OCP of the SS304 turned towards a positive value (≈265 mV) and that of the carbon steel A36 also reached a positive value (≈88 mV), as an indication that the “HB1” promoted the passivation of both steels. However, for the carbon steel, the OCP value may indicate an intermediate risk of corrosion.
5. After exposure to the “HB1” CE solution, SEM–EDS and XPS analysis suggested that the passive layer of SS304 was composed of ${\mathrm{Fe}}_{3}O_{4}$, ${\mathrm{Fe}}_{2}O_{3}$ and ${\mathrm{Cr}}_{2}O_{3}$, while ${\mathrm{Fe}}_{3}O_{4}$, FeO and ${\mathrm{Fe}}_{2}O_{3}$ were characteristic of the carbon steel A36 layer.
6. Probably due to the presence of ${\mathrm{Cl}}^{-}$ ions (originated from the pumice), isolated initial pits were observed in the vicinity of the carbides (local cathodes), causing a local depassivation. However, the preferential accumulation of ${{\mathrm{SO}}_{4}}^{2-}$ ions, with a higher charge than that of the ${\mathrm{Cl}}^{-}$ ions, may cause a retarding effect of the localized chloride attack over a longer time of exposure, which in fact agrees with the positive shift in the OCP.
7. The quantitative analysis of the EIS diagrams, based on the equivalent electrical circuit proposed, allowed us to characterize the corrosion activity of the studied steels at the metal–CE solution interface. Due to the passive state reached during the exposure to the “HB1” CE solution, both steels presented an increased $R_{p}$ value, being ≈4 higher for the SS304 than that of the carbon steel A36. The thickness of the passive layer formed on the SS304 surface was ≈1.8 nm, while that on the A36 was ≈0.3 nm. These differences could be attributed to the nature of the steels.
8. The reported results of this study, using the “HB1” CE solution to simulate the concrete–pore environment, suggest that the hybrid cement “HB1” may be considered as a “green” alternative for the partial replacement of PC with volcanic pumice. The corrosion behavior of the reinforcement steels will depend mainly on their nature and the composition of the concrete–pore environment.

## Figures and Tables

**Table 1:** Compositions (wt.%) of 304 austenitic stainless steel and A36 mild carbon steel, according to manufacturers.

Element (wt.%)	C	Cr	P	S	Mn	Si	Ni	Fe
304	0.08	18.00	0.045	0.03	2.00	1.00	8.00	Balance
A36	0.0650	0.02	0.004	0.0020	0.41	0.02	-	Balance

Note: Balance means “to complete the total 100%”.

**Table 2:** Composition (wt.%) of hybrid cement “HB1” [14].

HB1	CPC30R	${\mathrm{Na}}_{2}{\mathrm{SO}}_{4}$	${\mathrm{Ca}(\mathrm{OH})}_{2}$	Pumice (${\mathrm{SiO}}_{2}/{\mathrm{Al}}_{2}O_{3}$)	Total
g	300	36	18.78	300	654.78
wt.%	45.82	5.49	2.87	45.82	100

Note: Volcanic pumice contains (wt.%): $K_{2}O$ (4.24), ${\mathrm{Na}}_{2}O$ (3.9), CaO (3.78) [14].

**Table 3:** Composition (wt.%) of “SS1” supersulfated cement based on pumice, hemihydrated Ca-sulfate (Hh), CPC30 and CaO [48].

SS1	CPC30	CaO	Hh (CS) (${\mathrm{CaSO}}_{4}\cdot {1/2H}_{2}O$)	Pumice (${\mathrm{SiO}}_{2}/{\mathrm{Al}}_{2}O_{3}$)	Total
wt.%	6.90	6.90	34.48	51.72	100

**Table 4:** Oxide compositions (wt.%) of hybrid cement (HB1), supersulfated (SS1) and Portland cement (PC).

Cement	SiO_2_	Al_2_O_3_	Fe_2_O_3_	CaO	MgO	SO_3_	K_2_O	Na_2_O	Cl	LOI
HB1	43.05	8.85	2.79	29.78	0.75	4.87	2.88	4.35	0.05	2.63
SS1	38.22	7.82	1.63	26.07	0.49	19.46	2.98	2.00	0.06	1.27
PC	22.30	4.62	2.44	58.42	1.92	2.20	0.35	0.28	-	3.62

**Table 5:** Ionic composition (mg/L) of the hybrid “HB1” CE solution.

Element (mg/L)	Li	$K^{+}$	${\mathrm{Na}}^{+}$	${\mathrm{Al}}^{3+}$	${\mathrm{Ca}}^{2+}$	Si	${{\mathrm{SO}}_{4}}^{2-}$	Sr	${\mathrm{Cl}}^{-}$	${\mathrm{OH}}^{-}$
HB1	1.32	2480.20	17,358.90	0.37	210.00	4.39	10,115.00	9.74	172.00	1515.00
SS1	0.18	496.80	310.20	0.18	396.60	5.4	563.50	9.02	45.40	407.80
PC [49]	-	1373.58	420.71	-	256.51	-	-	-	-	958.8

Note: [OH^−^] was calculated from the pH.

**Table 6:** Change in time of the “HB1” CE solution’s pH and the steel sample’s OCP (vs. SHE) average values.

	Time (Days)	Initial	1	7	14	21	30
304	pH	12.99	12.92	10.48	9.61	9.18	9.07
	OCP vs. SHE (mV)	−204.899	−89.811	+128.126	+219.756	+245.307	+265.085
A36	pH	12.95	12.93	10.11	9.57	9.08	9.10
	OCP vs. SHE (mV)	−89.053	−24.845	+48.584	+67.456	+79.154	+88.18

**Table 7:** Stainless steel 304 EDS average analysis (wt.%) of the areas marked on the SEM images (Figure 1).

Days/wt.%		Fe	Cr	Ni	O	Na	Ca	S	K	Si	C	Cl
7	D1	5.93	-	-	30.88	7.69	5.20	8.49	16.95	24.86	-	-
	D2	4.11	-	-	34.08	18.65	1.52	21.98	7.85	11.80	-	-
	D3	64.15	17.75	7.30	5.62	1.65	-	-	-	2.71	0.83	-
30	G1	2.42	0.74	-	50.98	10.24	1.15	2.06	6.40	25.08	-	0.91
	G2	2.38	-	-	51.86	14.81	-	6.49	6.96	17.50	-	-
	G3	5.97	1.90	-	52.68	6.05	-	0.86	2.19	21.15	8.87	0.32

**Table 8:** Carbon steel A36 EDS average analysis (wt.%) of the areas marked on the SEM images (Figure 3).

Days/wt.%		Fe	O	Na	Ca	S	K	Si	C	Al	Cl
7	B1	11.19	43.76	16.19	6.80	16.60	2.16	0.19	3.11	-	-
	B2	83.53	7.10	3.43	0.46	0.51	-	0.98	3.98	-	-
30	F1	8.29	50.18	1.04	18.19	-	-	-	12.30	-	-
	F2	1.20	45.18	16.07	6.25	13.74	12.51	-	2.63	-	1.76

**Table 9:** EDS surface average analysis (wt.%) of stainless steel 304 and carbon steel A36 areas of interest as marked on the SEM images (Figure 5).

Steel/wt.%		Fe	Cr	Ni	Mn	Si	C
304	1	70.32	19.01	8.67	1.30	0.70	-
	2	65.45	19.00	8.23	1.36	0.60	5.35
A36	A	98.15	-	-	-	-	1.85
	B	95.33	-	-	-	-	2.54

**Table 10:** Fitting EIS parameters obtained for the stainless steel 304 and the carbon steel A36 exposed to “HB1” CE solution up to 30 days.

Steel	Days	R_s_ $k\Omega \;{\mathrm{cm}}^{2}$	R_ct_ $k\Omega \;{\mathrm{cm}}^{2}$	CPE ${\mu \mathrm{Ss}}^{n}{\mathrm{cm}}^{-2}$	n	R_p_ $k\Omega \;{\mathrm{cm}}^{2}$	χ^2^ $10^{-4}$
304	0	0.01	698	26.01	0.93	698	3.63
	1	0.01	3155	20.88	0.93	3155	3.92
	7	0.01	9024	15.05	0.91	9024	2.82
	14	0.02	12,900	14.85	0.91	12,900	4.54
	21	0.02	12,360	14.40	0.91	12,360	6.10
	30	0.02	12,380	13.83	0.91	12,380	6.50
A36	0	0.01	262	73.95	0.91	262	1.96
	1	0.02	1156	63.33	0.92	1156	2.06
	7	0.02	2226	56.45	0.92	2226	4.92
	14	0.03	4268	51.26	0.89	4268	10.44
	21	0.03	3268	59.07	0.89	3268	13.59
	30	0.04	3186	58.32	0.89	3186	12.32

## Data Availability

The data are available upon request from the corresponding author.
